# Neuropathic Pain: Biomolecular Intervention and Imaging via Targeting Microglia Activation

**DOI:** 10.3390/biom11091343

**Published:** 2021-09-10

**Authors:** Aijun Ji, Jinbin Xu

**Affiliations:** Department of Radiology, Washington University School of Medicine, 510 S. Kingshighway Blvd., St. Louis, MO 63110, USA; jajoffice@163.com

**Keywords:** neuropathic pain, microglia, neuroinflammation, intervention, imaging

## Abstract

Many diseases, including cancer, can lead to neuropathic pain (NP). NP is one of the accompanying symptoms of suffering in many conditions and the life quality of NP patient is seriously affected. Due to complex causes, the effects of clinical treatments have been very unsatisfactory. Many experts have found that neuron-microglia interaction plays an essential role in NP occurrence and development. Therefore, the activation of microglia, related inflammatory mediators and molecular and cellular signaling pathways have become the focus of NP research. With the help of modern functional imaging technology, advanced pre-and clinical studies have been carried out and NP interventions have been attempted by using the different pharmaceuticals and the extracted active components of various traditional herbal medicines. In this communication, we review the mechanism of microglia on NP formation and treatment and molecular imaging technology’s role in the clinical diagnosis and evaluation of NP therapies.

## 1. Introduction

In 1994, the International Association for the Study of Pain (IASP) defined NP as the pain caused by primary injury or dysfunction of the nervous system [1]. In 2011, the IASP redefined the definition to pain caused by injuries directly or diseases of the peripheral or central somatosensory system. NP is a common chronic pain condition that originates from direct or metabolic diseases and viral infections. Traumatic injury or chemotherapy-induced peripheral or central nervous system (CNS) injury can lead to this disorder, which influences the signal transduction mechanism of nerves in the dorsal root ganglion (DRG), spinal cord and brain tissue. NP patients mainly manifest with physical symptoms such as dysesthesias, paresthesia, spontaneous pain, allodynia, hyperalgesia and other various features [2]. A common type of NP caused by cancer is malignant neuropathic pain (MNP). This disease produces severe adverse consequences for these patients’ quality of life. 

In the CNS, glial cells account for about 70% of the total number of cells. Glial cells include oligodendrocytes, astrocytes, ependymal cells, Schwann cells, microglia and satellite cells. Microglia account for 5~20% of total glial cells [3]. The microglia, the resident macrophages, are not dormant cells in a resting state. They can detect environmental changes and cooperate with other cells/neurons to maintain homeostasis. Microglia respond quickly with morphological changes when stimuli affect the physiological homeostasis of the CNS: microglia numbers increase, volume increases, processes retract and become ramified. Activated microglia have two polarization types: M1-type (M1) and M2-type (M2), pro-inflammatory and anti-inflammatory, respectively. M1 microglia have a strong phagocytic ability and can produce many pro-inflammatory factors [4,5]. It is known that NP is related to a change in the proportion of microglia polarization types, M1/M2. As microglia are the critical cells in the pathogenesis of NP, selective inhibition of M1 or promotion of M2 microglia polarization has long been a subject for research and target for treatment of NP. 

NP is a complex problem in clinical pain treatment and NP incidence is very high [6,7]. The etiology and mechanisms of NP are more complex than traumatic pain. Cancers can easily induce nerve injury that leads to NP due to the compression of the nerve tissue, the toxic reaction from radiotherapies and chemotherapies, surgery and other symptomatic factors.

In recent efforts to advance the treatments of NP, experts have tried to control the pain by inhibiting activated microglia. Furthermore, in recent years, renewed attention has been paid to glial cells’ role in NP and in particular their role in the CNS [8]. In this communication, the effect of microglia on NP and the related interventions and specific imaging approaches are reviewed.

## 2. Mechanisms Related to Microglia Activation

After stimulation from harmful factors, microglia respond quickly and are activated. Activated microglia can induce and release nociceptive inflammatory mediators. The main targets of current pain research include chemokines (CC), interleukins (IL), colony-stimulating factors (CSF), tumor necrosis factors (TNF), the inducible isoform of nitric oxide synthase (iNOS) and cyclooxygenase 2 (COX-2). Furthermore, microglia can directly express specific neurotransmitter receptors related to pain, such as G protein-coupled receptors and ionotropic receptors. Receptor expression activates intracellular signal pathways and enables microglia to respond to changes in their microenvironment quickly and effectively. 

### 2.1. Major Inflammatory Mediators

#### 2.1.1. Chemokines (CCs)

Chemokines are critical inflammatory mediators between peripheral sensory neurons and glial cells. Chemokines function to regulate the migration and activation of white blood cells. These factors regulate the production and development of NP and are related to various CC receptor subtypes found in the CNS. CC ligand 2 (CCL2) binds to CC receptor 2 (CCR2) with the highest affinity [9,10], a receptor expressed on the microglia found in the spinal cord. CCR2 interact with other chemokines such as CCL7, CCL8 and CCL13. Some studies have shown that intraspinal injection of CCL2 induced extensive microglia activation in the ipsilateral spinal dorsal horn. CCR2 antagonists have been demonstrated to increase animal pain threshold effectively, which suggests that the binding of CCL2 and CCR2 is involved in the interaction between neurons and microglia and the maintenance of hypersensitization in NP [11,12].

C-X3-C motif chemokine ligand 1 (CX3CL1) mainly exists in CNS neurons, peripheral DRG neurons and primary afferent neuron endings. CX3CL1 receptors are primarily expressed in the microglia of the brain and spinal cord. CX3CL1 becomes soluble upon the action of protease cathepsin S (Cat S) secreted by microglia. When peripheral nerve injury occurs, microglia increase Cat S secretion. One finding showed that if a Cat S inhibitor was given to rats, CX3CL1 would return to a normal level and touch-induced pain would be inhibited [13]. CCL21 mainly exists in unmyelinated sensory neurons and can be transported to the terminals of primary afferent neurons through spinal dorsal root axons. Normal DRG neurons scarcely express CCL21, but peripheral nerve injury can trigger CCL21 expression. Mice deficient of CCL21 expression did not develop NP post spinal nerve injury, while supplemental CCL21 administration activated spinal microglia and induced persistent pain [14]. The specific antibody of CCL21 can significantly inhibit the aforementioned symptoms, suggesting that CCL21 plays a particular role in microglial activation and NP [10]. However, the mechanisms by which CCL21 acts directly or indirectly on microglia remain to be investigated.

#### 2.1.2. Tumor Necrosis Factor-α (TNF-α)

TNF-α is the most crucial pro-inflammatory factor in nerve injury and the inflammatory response. TNF-α expression remained low or none in normal conditions. However, after peripheral nerve injury, TNF-α promotes Na^+^ influx and decreased excitatory threshold by activating the p38-MAPK pathway in DRG neurons. In the spinal cord, TNF-α is involved in the regulation of the NP through glutamate receptors (NMDA, AMPA, etc.) and the regulation of glutamate receptors may be a potentially effective target for NP therapy [15]. The expression of TNF-α and NF- kappa β are directly or indirectly related. In the DRG and the spinal dorsal horn, TNF-α can activate NF- kappa B by binding to its receptor. After that, the expression of other pro-inflammatory factors is induced, causing a cascade reaction that leads to the production of pain. In addition, the expression of TNF-α can lead to the upregulation of its primary receptor TNFR1 [16,17], which inhibits the spontaneous action potential of GABA neurons and weakening the inhibitory regulation of GABA [18], thus producing the pain effect found in NP.

#### 2.1.3. Interleukin-1β (IL-1β)

IL-1β is another cytokine that plays a vital role in the regulation of NP. The production of IL-1β is closely related to the activation of glial cells. Stimulated by pathological pain, microglia in the spinal dorsal horn are rapidly activated and change in morphology and function. These changes result in the up-regulation of IL-1β, NMDAR and phosphorylation of NMDA receptors (NR1 and NR2B) [19], changes in these expressions enhance pain conduction and promote the formation of pain sensitivity [20]. Intrathecal injection of the IL-1β specific antibody can prevent pain allergic reaction induced by MMP-9, indicating that IL-1β is involved in the development of NP [21].

#### 2.1.4. Colony Stimulating Factors (CSFs)

CSFs are cytokines involved in the proliferation and differentiation of hematopoietic stem cells into specific leukocytes such as macrophages and granulocytes. M-CSF is mainly expressed in DRG neurons and its receptor is only expressed in microglia of the spinal cord. It has been found that M-CSF can act on M-CSF receptors to promote the activation and proliferation of microglia through the immunomodulatory adapter protein DAP12 located on the microglia membrane. Thus, due to the role of M-CSF in the regulation of NP, mice lacking the M-CSF gene could prevent the activation of microglia and the occurrence of NP after peripheral nerve injury [22,23].

#### 2.1.5. Inducible Nitric Oxide Synthase (iNOS)

iNOS is an essential rate-limiting enzyme for nitric oxide synthesis. Excessive levels of iNOS are known to activate microglia. Activated microglia in the spinal cord could produce reactive oxygen species, nitric oxide, and inflammatory mediators - all of which are involved in pain hypersensitivity. Thus, peripheral nerve injury could lead to the overexpression of iNOS in the spinal cord. Results have suggested that systemic or intrathecal injections of an iNOS inhibitor could have effective analgesic properties for NP in WT mice. In contrast, iNOS knockout KO mice did not show hyperalgesia or abnormal pain symptoms induced by nerve injury [24].

#### 2.1.6. Cyclooxygenase-2 (COX-2)

COX-2 is mainly expressed in the cell body, proximal dendritic process and distal dendritic spine in neurons of the CNS. In the peripheral nervous system, COX-2 is highly expressed in macrophages and Schwann cells. In the resting state, microglia only express a small amount of COX-2. However, under the stimulation of inflammatory substances such as lipopolysaccharides (LPS), the morphology of microglia changes and the COX-2 mRNA and protein expression increase rapidly. At the same time, these changes promote the release of neurotoxic substances: TNF-α, IL-1, IL-6 and NO which are known to damage neurons and consequently lead to NP [25].

### 2.2. Signal Pathways Related to Microglia

**Figure 1:**
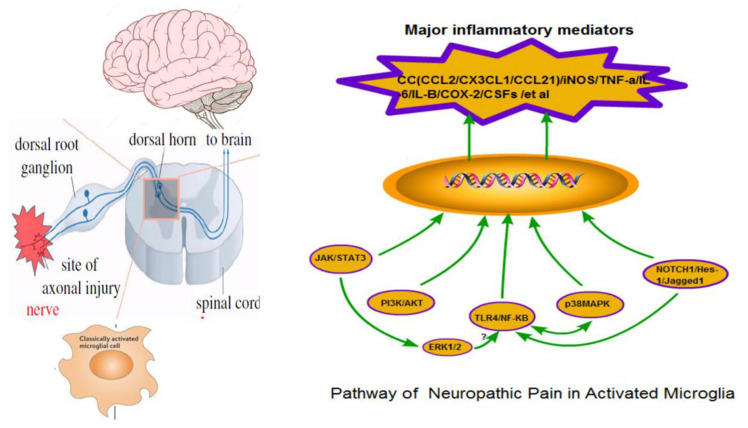
Neuropathic pain and microglia signal transduction pathway. DRG: dorsal root ganglia; CC: chemokines; IL: interleukin; CSFs: colony stimulating factors; TNF: tumor necrosis factor; iNOS: inducible nitric oxide synthase; COX-2: cyclooxygenase 2; p38MAPK: p38 mitogen-activated protein kinase; ERK: extracellular signal-regulated kinase; TLR4: toll-like receptor 4; NF-κB: nuclear factor-kappa B; PI3K: phosphatidylinositide 3-kinase; AKT: proteinkinaseB; JAK: c-Jun N-terminal kinase; STAT3: Signal Transducer In addition, Activator Of Transcription 3; NICD: notch intracellular domain); RBP-Jκ: recombination signal binding protein-Jκ; TACE: tumor necrosis factor-α converting enzyme.

At present, the neuroglia signaling pathways: JAK/STAT3, PI3K/AKT, MAPK, NF-κB and Notch, are relevant to the current understanding of NP (Figure 1).

#### 2.2.1. JAK/STAT3 Signaling Pathway

IL-6, IFN-γ, IL-10 and other cytokines can bind to their appropriate cytokine receptors and induce JAK phosphorylation of the receptor. Subsequently, STAT binds to the phosphorylated sites and JAK phosphorylates two STAT molecules to form a dimer that activates transcription of target genes in the nucleus. STAT3 in the STAT family plays a vital role in pain conduction and microglial activation [26]. STAT3 induces microglia to release inflammatory factors such as iNOS, IL-6 and CCL5 [27]. IL-6 induces activation of the JAK/STAT3 signaling pathway and promotes the release of iNOS, IL, TNF-α and CCL2. The JAK/STAT3 signaling pathway is associated with microglia proliferation and is involved in microglia polarization [28].

#### 2.2.2. PI3K/AKT Signaling Pathway

The PI3K/AKT pathway is an intracellular signal transduction pathway involving PI3K and AKT. Nociceptive chemokine CCL2 can activate microglia and the PI3K/AKT pathway, which leads to pain hypersensitization and participates in the occurrence and development of NP. However, some studies have also shown that PI3K/AKT signaling pathway activation mainly occurs in the early stage of NP. In addition, PI3K/AKT regulates synaptic plasticity and long-term potentiation (LTP) [29] and the application of PI3K/AKT inhibitors reduced the microglia activation, decreased the expression nerve growth factor (NGF) and relieved chronic neuronal pain [30]. On the other hand, the PI3K/AKT signaling pathway had a negative regulatory mechanism. PTEN is a tumor inhibitor gene, which could inhibit the PI3K/AKT signaling pathway [31].

#### 2.2.3. MAPK Signaling Pathway

Three signaling pathways, p38 mitogen-activated protein kinase (p38MAPK), extracellular signal-regulated kinase (ERK) and c-Jun N-terminal kinase (JNK), are mainly involved in the regulation of NP. P38MAPK is the crucial intracellular signaling molecule that regulates the pain signals of microglia. The phosphorylation of p38MAPK occurs in microglia. The expression of TLRs, P2X4R, P2X7R, P2Y12/13R and other membrane receptors is upregulated after the activation of spinal cord microglia [32]. The phosphorylation of p38 MAPK can increase the expression and synthesis of inflammatory factors and chemokines, promote the formation of central hyperalgesia of pain and facilitate pain signal transduction [33]. It has been proved that intrathecal injection of p38MAPK inhibitor SB230580 into an animal model of neuronal pain can reduce the pain sensation [34]. Still, a p38MAPK inhibitor can only prevent the occurrence and development of chronic NP, not reverse the pain symptoms. The mechanism of SB230580 is still unclear. ERK is an extracellular signal-regulation protease of the MAPKs family. ERK1/2 phosphorylation is closely related to NP pain-sensitive changes and appears in microglia a few days after nerve injury. Increased ERK1/2 phosphorylation is accompanied by up-regulation of inflammatory factors IL-1 β and IL-6 as well as TNF-α at the mRNA level. Inhibition of ERK can relieve mechanically tactile induced pain and weaken the central sensitization of the spinal dorsal horn [35,36,37]. JNK is known as s stress-activated protein kinase (SAPK). It has been suggested that the JNK signaling pathway is mainly involved in the maintenance of NP [38]. MAPK (ERK1/2 and JNK) may act as upstream signaling molecules for NF-κB activation. If the JNK pathway is suppressed, then NF-κB expression could be decreased, leading to the down-regulation of IL-6 and TNF-α at the transcription and translation levels, respectively [39].

#### 2.2.4. TLR4/NF-κB Signaling Pathway

NF-κB is a nuclear transcription factor in B lymphocytes that are involved in the regulation of many cytokines. In the nervous system, NF-κB only exists in two subtypes of p50 and p65. NF-κB mainly regulates the inflammatory response through classical and non-classical pathways. Cytokine receptors and toll-like receptors (TLR) are involved in the classical pathways. TLRs are pattern recognition receptors primarily expressed in glial cells in the rodent CNS. In a NP animal model of nerve injury, mechanical hyperalgesia and thermal hyperalgesia are closely associated with TLR4 overexpression in the spinal cord microglia. [40,41]. Inhibiting the expression of TLR4 could reduce the activation of microglia, the release of inflammatory mediators and lessen the hyperalgesia. TLR4/NF- kappa B is a non-Ca^2+^ dependent pathway of pain signal transduction. When peripheral nerve injury occurs, nociceptive transmitters combine with TLR4 and activate the receptor, NF-κB, downstream related factors and microglia. This cascade promotes the synthesis and release of IL-6, IL-1β, TNF-α, iNOS and COX-2 to sensitized spinal dorsal horn neurons, which ultimately causes NP [42,43]. Thus, inhibition of the TLR4/NF- kappa B pathway may be one of the most effective strategies for anti-inflammatory therapies of NP.

#### 2.2.5. Notch Signaling Pathway

The Notch signaling pathway is comprised of Notch receptors, Notch ligands (DSL protein), NICD transport molecules and downstream Hes/Hey [44]. The Notch signaling pathway plays an essential role in the activation and proliferation of glial cells. LPS can activate microglia in a resting state. Activated M1 microglia express a large number of Notch1, Notch2 and ligand Jagged1. Downstream target genes Hes-1 and RBPJ kappa release many inflammatory mediators that significantly decrease pain threshold, such as IL-6, TNF-α, IL-1β and iNOS [45,46]. After blocking the Notch signaling pathway with DAPT, M1 cells reduce inflammatory mediators and evolved into M2 cells. Inhibition of the Notch signaling pathway can significantly impede microglia activation, thus, decreasing the expression of pro-inflammatory cytokines and in turn reduce the injury to neurons and reverse the hyperalgesia. In addition, NF-κB, as the downstream factor of the Notch signaling pathway, also plays an essential role in regulating the expression of IL-1β, IL-6, TNF-α, iNOS and other inflammatory mediators [47]. Whether the Notch signaling pathway could activate microglia directly or indirectly and whether it interacts with different pathways remains to be confirmed by further studies.

## 3. Intervention Studies Related to Microglia

Focusing on the mechanism of microglia activation and NP, the purpose of this study was to reverse NP by discovering microglia inhibitors or blocking related signaling pathways. At present, the research on drug therapies for NP is mainly focused on the mechanism and clinical efficacy of the combined use of third-line drugs and drug and non-drug cocktails. The scope of current research aims to increase the therapeutic effectiveness and reduce adverse reactions to NP. Some research is carried out at the molecular level. As an inhibitor of microglia activation, minocycline (MI) reduces the expression and release of pain-causing substances such as IL-1, TNF-α and CX3CL1. MI directly acts on ion channels and inhibits the phosphorylation of intracellular kinases such as MAPKs and thus could effectively alleviate NP. Edaravone may inhibit the activation of the Notch signaling pathway and the NF-kappa B signaling pathway, PI3K/Akt. The regulated expression of NF-kappa B could decrease the release of related inflammatory mediators. Glial inhibitor propento-fylline (PPF), a methylxanthine derivative, can inhibit the activation of glial cells, antagonize the release of active substances and inhibit the release of inflammatory factors nitric oxide and other related substances [48,49,50]. However, there is no definite report on whether these chemicals have been used and how effective they may be. Studies in animal models have explored the molecular signal pathways and related inflammatory mediators of NP by utilizing biologically active molecules extracted from traditional herbal medicines known to produce analgesic effects.

### 3.1. NF-Kappa B Signaling Pathway

Curcumin is the main biologically active component extracted from turmeric roots. Curcumin could inhibit the activation of NF- kappa B and down-regulate the expression of Toll-like receptor4 and spinal cord CX3CR1 by down-regulating the activities of nuclear factor kappa-B kinase (Ik-Bk) and protein kinase B (Akt) inhibitors. Some studies have also shown that curcumin could inhibit microglia activation and reduce the release of LDH, TNF-α, IL-1β and IL-6 by inducing LPS through the JAK2 / STAT3 signaling pathway, thereby controlling the formation of NP. Tetramethypyrazine (TMP) is an alkaloid isolated from Ligusticum chuanxiong. Ligustrazin can increase the mechanical pain threshold and the thermal claw latency in rats and has a pronounced analgesic effect. Resveratrol could effectively relieve thermal pain and mechanical pain, especially in severe pain, by inhibiting the activation of the NF-kappa B signaling pathway and decreasing the expression of NF-kappa B and the release of many inflammatory cytokines such as TNF-α. Catalpol is a small molecule compound of iridoid extracted from the root of Rehmannia glutinosa. Studies have shown that catalpol could regulate TNF-α and Akt, inhibiting the NF- kappa B signaling pathway, thus inhibiting the activation of microglia and reducing the release of inflammatory factors [51,52,53,54,55].

### 3.2. MAPK Signal Pathway

Tanshinone IIA, the main active component of Salvia miltiorrhiza, can effectively inhibit the spinal cord microglia activation in the spinal nerve ligation (SNL) animal model. The mechanism is related to the inhibition of MAPKs activation and the resulting decreased expression of TNF-α and IL-1β. *Polygonum cuspidatum* extract has a significant inhibitory effect on mechanical hyperalgesia induced by CCI and its mechanism may be related to the inhibition of p38 phosphorylation in spinal cord microglia. Procyanidins (PC) is a natural flavonoid existing richly in grape seeds, blueberries, cherries and other plants. PC could improve opioid-induced hyperalgesia (OIH) by inhibiting microglia activation and inhibiting the pd38MAPK pathway, reducing the response of LPS induced inflammation [56,57,58]. *Tripterygium wilfordii* could inhibit microglial activation, the phosphorylation of MAPKs and the overexpression of inflammatory cytokines such as IL-6, IL-1β, TNF-α and Monocyte Chemotactic Protein (MCP-1). *Gastrodin* could be effectively used in the treatment of NP as it inhibits vincristine-induced hyperalgesia in rats. Its mechanism may be related to the inhibition of ERK1/2 and the activation of spinal microglia [59,60,61].

### 3.3. Other Inflammatory Mediators

Corydalis, extracted from the tuber of the poppy family, is commonly used in traditional herbal medicine for relieving pain. *Elaeagnus angustifolia* contains a variety of alkaloids among which, tetrahydropalmatine B has the most potent analgesic effect. It has been shown to increase the pain threshold of chemotherapy pain rat models, which may be related to its inhibition of the expression of TNF-α, IL- 1β and other inflammatory cytokines in the lumbar segment of the spinal cord [62]. *Iridoid glycosides* could significantly relieve mechanical tactile pain and temperature hyperalgesia via decreased NOS activity and NO levels as well as inhibition of iNOS mRNA expression in the spinal cord [63]. Both hookitin and artemisolactone were shown to reduce the production of inflammatory factors TNF-α, IL-1β and IL-6 in microglia induced by LPS and effectively relieve NP hyperalgesia. Cinobufagin could significantly relieve mechanical pain and thermal pain sensitivities in rats by inhibiting the activation of rat spinal cord microglia and reducing the release of TNF-α, IL-1β and other related inflammatory mediators by spinal cord glial cells.

The current systematic and comprehensive research of NP medicine is still preliminary. Generally speaking, the experimental study of the active components in traditional herbal medicine and the analgesic mechanisms of microglia is dispersed and insufficient. Thus, questions remain to be answered, such as whether the active ingredients of traditional herbal medicine can be directly applied to the clinical treatment of NP, whether the combination of different treatments for different NP targets has a synergistic effect and whether a more effective procedure can be developed to minimize the side effects caused by existing NP therapies.

## 4. Neuroimaging of Microglia Activation

Many imaging technologies had been used as new research methods such as non-invasive or less invasive positron emission tomography (PET), single-photon emission computed tomography (SPECT), magnetic resonance imaging (MRI) and other neuroimaging techniques to study the relationship between the occurrence and developmental mechanisms and imaging of chronic pain, including NP. As a result, researchers can now observe the activation process of glial cells dynamically and quantitatively in vivo and develop new clinical diagnostic procedures and more effective evaluations of NP treatments [64].

### 4.1. Imaging via Targeting Opioid Receptor

Opioid receptors are the most central receptors to the study of pain and analgesia. Quantitative detection of μ, σ and κ opioid receptors and their signal transduction in the brain could provide direct insight into pain mechanisms. PET tracers such as [^11^C]DPN, [^11^C]carfentanil and [^11^C]Diprenorphineand and SEPCT tracers such as [^123^I]DPN and [^123^I]-o-IA-DPN have been widely used in functional imaging techniques. These tracers could show regional differences in opioid receptor densities and subtype distributions, providing methods for the dynamic study of opioid receptor distribution and changes under physiological and pathological conditions [65].

In chronic pain, endogenous opioid peptides are released, vandalized and downregulated. Subsequently, the binding affinity of opioid receptors to radiotracers also decreases. The motor cortex stimulation (MCS) for NP control studies showed that the binding of [^11^C]DPN in the frontal cortex, anterior middle cingulate cortex (aMCC) and periaqueductal gray (PAG) is significantly decreased. The binding changes in aMCC and PAG substantially correlate with the degree of pain relief, indicating that MCS could induce the release of endogenous opioid peptides from the brain to attenuate chronic pain [66]. However, because various factors regulate the opioid receptor activation and its binding state to ligands, opioid receptor PET imaging alone may not fully reveal the complex nature of this receptor and its changes throughout the NP developments and interventions.

### 4.2. Imaging via Targeting Translocator Protein

Translocator protein 18 kDa (TSPO), also known as peripheral benzodiazepine receptor (PBR), is well characterized as a biomarker of neuropathological changes in vitro and in vivo. The expression of TSPO in resting microglia is low, but it is upregulated in activated microglia, suggesting that TSPO may be involved in microglia activation and could serve as a biomarker for neuroinflammation. PET imaging studies using TSPO radiotracers [^11^C]PK11195 and [^11^C]PBR28 have shown that the uptake of both tracers is significantly higher in the patients suffering from pain compared to controls in the thalamus, anterior and posterior central gyrus and the paracentral lobule. This finding suggests that the activation of human microglia is pain-related, as is the secretion of inflammatory mediators such as TNF-α, IL-1β, IL-6 and COX-2 [67,68]. After activation of TSPO by endogenous or exogenous ligands, the transportation of TSPO-dependent cholesterol to mitochondria is accelerated and neuro-steroid production is increased. Some TSPO ligands, such as Ro5-4864, etifoxine, AC 5216 and MPIGA, have been shown to mediate the increase of neuro-steroid hormone production. Ro5-4864 had neuroprotective and anxiolytic effects and effectively attenuated the anxiety and other related symptoms caused by nerve injury [69,70]. In a rat model of the development of NP, TSPO PET-CT imaging of the lumbar spinal cord showed decent aggregation of [^11^C]PK11195 in conjunction with the glial cell increases in the spine [71]. These studies suggest that TSPO PET might be valuable for evaluating the glia activation involved in NP.

#### 4.2.1. PBR28 Binding Affinity

**Figure 2:**
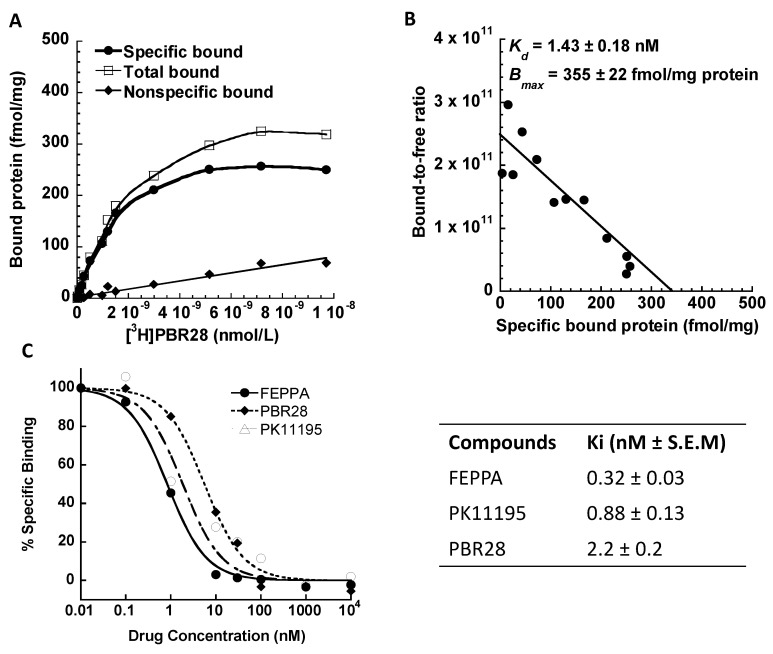
Scatchard analysis and pharmacological profile of [^3^H]PBR28 binding to PBR in rat brain membrane homogenates. (**A**): Representative saturation binding experiment which shows the total bound, nonspecific bound and specific bound. (**B**): Representative Scatchard plot of the *K*_d_ (1.43 ± 0.18 nM) and *B*_max_ (355 ± 22 fmol/mg protein). (**C**): Representative competitive binding data for inhibition of [^3^H]PBR28 binding to PBR in rat brain by known PBR ligands FEPPA (*K*_i_ = 0.32 ± 0.03 nM), PK11195 (*K*_i_ = 0.88 ± 0.13 nM) and PBR28 (*K*_i_ = 2.2 ± 0.2 nM). The values are the mean ± S.E.M for n = 3 determinations, samples in duplicate.

To measure the TSPO binding affinity of PBR28 and the TSPO receptor density in the brain tissue, [^3^H]PBR28 was synthesized by American Radiolabeled Chemicals, Inc. (St. Louis, MO, USA). The specific activity of the radioligand was 80 Ci/mmol. Receptor binding studies, both saturation and competitive assays were carried out with rat brain tissue homogenates as reported [72,73]. The data showed that this radioligand has a very high binding affinity with a dissociation constant *K*_d_ = 1.4 nM, high maximum receptor binding sites *B*_max_ = 355 fmol/mg protein and low nonspecific binding to TSPO in the rat brain membrane homogenates. Known TSPO selective small-molecule ligands, FEPPA, PK11195 and PBR28, displaced the binding sites with high affinity, expressed as dissociation constants *K*_i_ of 0.32 nM, 0.88 nM and 2.2 nM, respectively (Figure 2A–C).

#### 4.2.2. TSPO Postmortem Autoradiography in Human Brains

**Figure 3:**
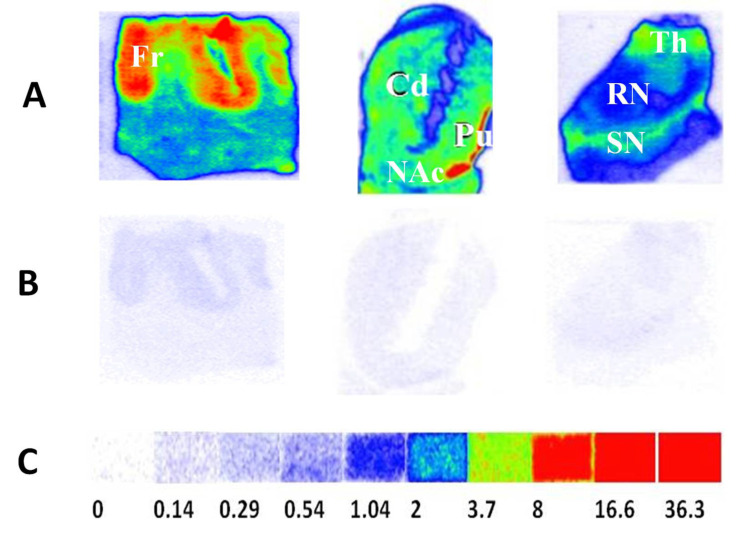
The striatal and extra-striatal TSPO distribution in the human brain. Autoradiograms show the total binding of 2 nM [^3^H]PBR28 (panel **A**) in the frontal cortex, striatum and substantia nigra regions of human brain sections. Nonspecific binding of the radiotracer was determined using adjacent tissue sections with 1 μM PK11195 to mask TSPO (panel **B**). [^3^H]Microscale standards (ranging from 0 to 36.3 nCi/mg) were also counted for the calibration of radioactivity (panel **C**). The following CNS anatomical regions are denoted: Frontal cortex (Fr); Putamen (Pu); Caudate (Cd); Nucleus accumbens (NAc); Thalamus (Th); Substantia nigra (SN); Red nucleus (RN). For autoradiograms: red indicates high, blue indicates medium and white indicates low binding activity. Adapted from Xu et al., 2019 [74].

In a previous study, the TSPO alterations were investigated in the advanced Alzheimer disease (AD), dementia with Lewy bodies (DLB) and Parkinson disease dementia (PDD) brains via quantitative autoradiography using [^3^H]PBR28 as the radioligand. [^3^H]PBR28 turned out to be an excellent radiotracer for autoradiography measure of TSPO densities in postmortem human brains with high specific binding and negligible nonspecific binding. The TSPO density was unchanged in the frontal cortex, striatum, thalamus and red nucleus of DLB/PDD brains. However, a significant reduction in TSPO density was found in the substantia nigra (SN) of DLB/PDD brain compared to age-matched healthy controls (Figure 3). This distinct change pattern of TSPO density in the late-stage DLB/PDD cases may imply the occurrence of microglia dystrophy, senescence, or death in late-stage neurodegeneration [74]. TSPO, a microglia activation marker in the early stage of AD and DLB/PDD, may also be used to monitor the microglia dysfunction in the late-stage disease. Recent postmortem autoradiography studies also showed that [^3^H]PBR28 is a valuable neuroinflammation probe for studying microglia engagement in AD tauopathies and disease progression of AD and Lewy body diseases (LBDs) [75,76]. Notably, the first human TSPO blocking studies showed that [^11^C]PBR28 binding in brain can be blocked by a known TSPO ligand XBD173, which confirmed this radiotracer’s selectivity and specificity for imaging TSPO in vivo.

To date, emerging PET probes have been developed to specifically target microglia and astrocytes using a novel colony-stimulating factor 1 receptor (CSF1R) tracer [^11^C]CPPC [77] and an imidazoline-2 binding sites (I_2_BS) radioligand [^11^C]BU99008, respectively [78,79]. TSPO probes, together with these novel human glia PET tracers, will benefit the NP research community by aiding to dissect the individual roles of glia subtypes in pain sensation.

### 4.3. [^18^F]FDG PET Imaging

In a recent clinical investigation, [^18^F]FDG PET/MRI showed promises in locating hypermetabolic abnormalities in the non-brain brain regions of NP patients [80]. Furthermore, earlier [^18^F]FDG PET/CT imaging studies of carcinomatous NP showed there are decent radioactivity uptakes in the nodular, fasciculate and root whisker along the nerve root and bundles in peripheral nerves with metastases or tumor invasion; suggesting that [^18^F]FDG PET/CT could measure the density, distribution and structure of nerves involved in patients with carcinomatous NP pain. However, the influence of iatrogenic factors such as puncture, surgery, radiotherapy and chemotherapy, etc. on the nerve SUVmax needs to be excluded [81,82].

## 5. Conclusions and Prospect

The pathogenesis of NP is complex, but many studies have confirmed that microglia activation is involved in the development of NP. The microglia activation, up-regulation of specific membrane receptors, enhancement of the intracellular signaling pathways and the release of pain-related inflammatory mediators are the pathophysiological basis for the occurrence and development of NP. Although these studies are limited to cell and animal experiments, these beneficial explorations have deepened our understanding of NP. At present, drugs are still an essential means of NP treatment and antidepressants and anticonvulsants are the first-line drugs for NP treatment and opioid analgesic drugs have a synergistic effect. In addition, medications such as nerve blocks, electrical stimulation, intrathecal analgesia pumps, nerve injury and other non-drug therapies also play essential roles in alleviating NP. In addition, psychological and behavioral interventions play a positive role in auxiliary treatments. However, outstanding problems remain, such as the difficulties in reversing pain sensitivity, significant individual differences in curative effect, drug tolerance, noticeable side effects, etc. As the symptomatic effects of NP are not ideal, these are still complex and thorny problems in global pain treatment. The modern molecular imaging technology via targeting TSPO using PBR28 or other specific biomarkers of microglia and astrocytes activation will advance the clinical diagnosis and evaluation of the NP pharmaceutics. Furthermore, more potent radiotracers specifically targeting microglia or astrocytes will be needed for further understanding the cellular mechanisms of glia activation in NP and accelerate the finding of effective interventions that target glia activation.

## Data Availability

All data have been included in this communication.
